# Titanium Pyrophosphate for Removal of Trivalent Heavy Metals and Actinides Simulated by Retention of Europium

**DOI:** 10.1155/2017/2675897

**Published:** 2017-07-12

**Authors:** Huemantzin Balan Ortiz-Oliveros, Rosa María Flores-Espinosa, Eduardo Ordoñez-Regil, Suilma Marisela Fernández-Valverde

**Affiliations:** ^1^Dirección de Investigación Tecnológica, Instituto Nacional de Investigaciones Nucleares, A. P. 18-1027, Col. Escandón, Delegación Miguel Hidalgo, CP 11801, Ciudad de México, Mexico; ^2^Universidad Autónoma del Estado de México, Instituto Literario 100, CP 50000, Toluca, Estado de México, Mexico; ^3^Dirección de Investigación Científica, Instituto Nacional de Investigaciones Nucleares, A. P. 18-1027, Col. Escandón, Delegación Miguel Hidalgo, CP 11801, Ciudad de México, Mexico

## Abstract

This work addresses the synthesis of titanium pyrophosphate, as well as the characterization and evaluation of the sorption process of europium, for removal of trivalent heavy metals and actinides simulate. The evaluation of the surface properties of titanium pyrophosphate was carried out determining the surface roughness and surface acidity constants. The values obtained from the determination of the surface roughness of the synthesized solid indicate that the surface of the material presents itself as slightly smooth. The FITEQL program was used to fit the experimental titration curves to obtain the surface acidity constants: log⁡*K*^+^ = 3.59 ± 0.06 and log⁡*K*^−^ = −3.90 ± 0.05. The results of sorption kinetics evidenced that the pseudo-order model explains the retention process of europium, in which the initial sorption velocity was 8.3 × 10^−4^ mg g^−1^ min^−1^ and kinetic constant was 1.8 × 10^−3 ^g mg min^−1^. The maximum sorption capacity was 0.6 mg g^−1^. The results obtained from sorption edge showed the existence of two bidentate complexes on the surface.

## 1. Introduction

The rapid growth of our population during the last decades has led to an ecological imbalance in our environment with severe consequences and major risks both for our health and our environment. An example for this is the cases of contamination of hydric resources. The most common water pollutants are fecal residues from animals and humans, pesticides used in agricultural activities, organic residues derived from oil, heavy metals, and radionuclides, among others [1–4]. In recent years, persistent organic contaminants such as phenols, chlorophenols, chlorobenzenes, and pharmaceutical drugs [2], as well as heavy metals like As, Cd, Cu, Cr, Pb, Hg, and so forth, or radionuclides like Ra and actinides, are becoming more common in hydric resources. These have caught the interest of the scientific community due to their high toxicity, easy dispersion, bioaccumulation, and persistency in the environment [5–9], particularly for actinides, whose long half-life and high radiotoxicity pose a health risk since they can lead to various diseases such as poisoning, nervous system damage, and cancer [10]. The toxicity and behavior of persistent organic compounds depend on their concentration, molecular structure, and type of functional groups. Also, heavy metals can react with organic matter forming organometallic compounds, which may be more toxic to aquatic ecosystems [11].

The contamination of hydric resources with metals and radionuclides is linked to manufacturing processes, the metal-mechanic industry, oil processing, mining, transportation processes of erosion material, infiltration of subterranean waters, and nuclear accidents, among others [12, 13].

Due to the chemical properties of these metals, their removal requires specific treatment processes. The processes most used are chemical precipitation, coagulation/flocculation, flotation (with dispersed air or dissolved air), electrochemical process, photocatalytic process, inverse osmosis, ion exchange, and sorption [14–20]. Many of these processes are usually efficient but can be limited by the concentration and the physicochemical form of the heavy metal as well as the costs and the difficulties of the operation such as the generation of residual sludge.

Sorption is one of the most used processes for the removal of low concentrations (including trace levels) of heavy metals and radionuclides in an aqueous medium. The term sorption is used in a generic way to describe physicochemical processes in which the dissolved contaminant (metal, radionuclide, etc.) is transferred from the solution to the solid phase [21, 22], either by precipitating on the surface, diffusing on the outside and inside of the pores of the solid phase, or by forming complexes on the surface [23].

The phenomena of sorption are also used with success in the construction of engineering barriers to avoid the dispersion of heavy metals and radionuclides in the soil and subsoil, where the sorption process is carried out by interaction of chemical species (metals or radionuclides) in solution with surface functional groups of the components of the soil [24, 25].

Due to the importance that sorption processes have in the treatment of industrial wastewater, in industrial applications or the remediation of soils contaminated with metals, interest has again risen during the last couple of years in carrying out investigations aimed at understanding the phenomena involved in sorption and also the development of different materials or sorbents and in determining their retention properties. In that regard, several investigators have studied low cost materials such as biosorbents, in which there is the biomass of residues of vegetable or animal origin or bacteria and fungi [8, 26, 27]. These biosorbents have proven themselves efficient in the removal of metals in aqueous solutions. In addition, new mineral sorbents have been synthesized and tested that unlike the biosorbents may cost more but have a high retention capacity, low solubility, and high thermic and structural stability, even when submitted to intense fields of gamma radiation, an example of which are the tests of sorption of metals and radionuclides in phosphates of isomorphic tetravalent metals, perovskite, and other synthetic inorganic materials [28–31].

As far as phosphates are concerned since the discovery of LiFePO_4_, new materials have been synthesized and identified based on phosphates-based polyanions such as (PO_4_)^3−^, (P_2_O_7_)^4−^, or (P_3_O_10_)^5−^ [32], which find their application in different fields of knowledge due to their major structural anomalies, such as their anisotropic deformation, low redox potential, and low cost of synthesis [32, 33]. These characteristics have motivated the study of the retention of metals in phosphates. The sorption of lanthanides and uranyl on different synthetic phosphates has been reported; for example, Wang et al. [34] studied the sorption of U (VI) onto Zr_1−*x*_Ti_*x*_P_2_O_7_ and TiP_2_O_7_; Ortiz-Oliveros et al. [35] studied the synthesis of *α*-Ti(HPO_4_)_2_H_2_O and sorption of Eu^3+^; and Maslova et al. [36] studied the synthesis and sorption properties of amorphous titanium phosphates.

Literature reports different synthesis methods of polyanions such as (P_2_O_7_)^4−^. The methods most used are (a) methods of coprecipitation where organic compounds are used as ion sources and phosphoric acids are used as phosphate sources [33, 34] and (b) hydrothermal methods where metallic oxides are used as ion sources and water-soluble phosphate salts as phosphate sources in an acid medium [32]. Rai et al. [33] used the method of coprecipitation to obtain titanium pyrophosphate. During this method, an aqueous solution of titanium isopropoxide is mixed with a solution of phosphoric acid and diluted hydrochloric acid. The mixture is heated to 343 K and agitated for 10-11 h. In the end, the obtained material is heated to 1073 K for 3 h in a CO_2_ atmosphere. Other authors obtain TiP_2_O_7_ using the sequence proposed by Patoux. It consists in mixing TiO_2_ and (NH_4_)_2_HPO_4_ and heating the mixture progressively to 1273 K using intermittent gridding sequences [45, 46].

This work addresses the synthesis of titanium pyrophosphate using an improved method. Titanium pyrophosphate was prepared using high purity TiCl_4_ and concentrated H_3_PO_4_, through precipitation in an atmosphere of nitrogen. The precipitate obtained was heated to 1073 K for 3 h. With titanium pyrophosphate previously characterized, the retention capacity of Eu^3+^ was evaluated as a chemical analogous for heavy metals (Cr^3+^ and As^3+^) and actinides (Ac^3+^) [47, 48]. It is well known that the europium ionic radius is similar to Cr^3+^, As^3+^, and Ac^3+^ radii, which results in a similar physicochemical behavior [49]. Additional, modeling the sorption of the europium onto the titanium pyrophosphate as a function of the pH using a surface complexation model was performed.

## 2. Experimental

### 2.1. Synthesis of TiP_2_O_7_

The titanium pyrophosphate was obtained by the described method by Ortiz-Oliveros et al. [35]. Synthesis was performed mixing high purity liquid titanium tetrachloride (99.9%, Aldrich) as the ion source with concentrated phosphoric acid (85%, Baker) as the phosphate source, while maintaining a Ti/P stoichiometric ratio of 1 : 2 under a nitrogen atmosphere in a glovebox. TiCl_4_ was slowly added to a stirred solution of phosphoric acid, and the precipitate was recuperated and washed with deionizer water. The precipitate was then centrifuged, separated, and dried at room temperature. Finally, the dried solid was heated at 1073 K for 3 h.

### 2.2. Material Characterization

The solid obtained was characterized by X-ray powder diffraction (XRD) patterns in a diffractometer (Siemens D5000). The XRD patterns were obtained with Cu monochromatic K*α* rays at 35 kV and 25 mA. The 2*θ* diffraction angle (4°–70°) was scanned at a scan rate of 1.83 min^−1^. The elemental and structural analysis of the solid was performed in a scanning electron microscope (PHILIPS model XL-30), coupled to a microprobe (EDAX model DX-4) for energy dispersive X-ray spectroscopy with a resolution of 140 eV. Representative samples were fixed on metal slides with carbon tape and were covered with a thin film of conducting material (Au).

Additional, samples of titanium pyrophosphate before and after europium sorption were investigated by Atomic Force Microscopy (Cypher Asylum Research Microscope).

Finally, the specific area (*A*_*s*_) was determined in a GEMINI 2360 Micrometrics surface area analyzer.

### 2.3. Determination of Surface Properties

The surface properties were evaluated using analytical grade reagents: potassium nitrate (Sigma-Aldrich ≥ 99%) and potassium hydroxide (Baker ≥ 85%). All of the solutions were prepared with deionized water under a nitrogen flux, and potassium nitrate was chosen as the background salt for fix the ionic strength. At the same time, all potentiometric titrations were performed in a potentiometer ThermoOrion 720A+ with a combined Ag/AgCl electrode.

The evaluation of the surface properties of titanium pyrophosphate was carried out determining (a) the surface roughness and (b) the surface acidity constants (*K*^+^ and *K*^−^).

*Surface Roughness.* The characterization of the superficial irregularity of the material (roughness) was determined according to Ismail and Pfeifer [50]. This method consists in determining the surface fractal dimension by means of N_2_ adsorption isotherms and (1):

(1) $$\begin{array}{c} \ln\left(\;\frac{V}{V_{m}}\;\right)=\Gamma +A\left[\;\ln\left(\;\ln\left(\;\frac{P_{o}}{P}\;\right)\;\right)\;\right], \end{array}$$

where *V* represents the volume of adsorbed gas molecules (mL g^−1^) at the equilibrium pressure (*P*) and saturation pressure (*P*_*o*_); *V*_*m*_ is the volume of monolayer coverage in mL g^−1^;* Γ* is an exponential factor; and *A* is a power-law exponent dependent on the fractal dimension (*D*) and adsorption mechanism. The N_2_ adsorption isotherm was determined in a GEMINI 2360 Micrometrics surface area analyzer.

*Surface Acidity Constants*. The surface acidity constants were determined by fitting the experimental potentiometric titration curves using the FITEQL 4v program [51, 52]. This computer program determines the chemical equilibrium constants from experimental data obtained by potentiometric titrations. The equilibrium model must be solved at each titration point; therefore, the parameters are adjusted to minimize the difference between the experimental values and those calculated from the model. Several Surface Complexation Models have been used to simulate the experimental data, and in this case, the Constant Capacitance Model (CCM) was used to fit the potentiometric titrations curves. This model has been used in many solids such as hydrous ferric oxides, phosphates, and titanates. Our experimental conditions (0.5 M) meet the ionic strength restriction in this model, and this model is preferred because of the relatively low number of adjustable parameters. These parameters in the CCM are *K*^+^ and *K*^−^ constants, the inner-layer capacitance (*C*), the total concentration of surfaces sites, and *A*_*s*_.

### 2.4. Sorption Experiments

All of the sorption experiments were carried out in polypropylene tubes at 303 K and under a nitrogen atmosphere with 400 mg of titanium pyrophosphate for 10 mL of aqueous solution (1 × 10^−4 ^M of europium nitrate, Aldrich 99.9%). Prior to the sorption experiments, the solid was first hydrated with 0.5 KNO_3_ solutions for 24 h and stirred at 45 rpm, which was shown to be sufficient to reach equilibrium. Afterward, the suspensions were centrifuged at 3500 rpm for 15 min, and all of the supernatant was removed.

A 10 mL aliquot of the europium nitrate stock solution was added to the hydrated solid and then the suspensions were adjusted to the required pH values and contact time, respectively.

The experiments carried out were (1) the evaluation of sorption kinetics and (2) modeling the sorption as a function of the pH using a surface complexation model. For the sorption kinetics of Eu^3+^, sorption isotherms were performed as a function of contact time by estimating the Eu(NO_3_)_3_ concentrations retained in the titanium pyrophosphate at different contact times (1, 3, 5, 7, 16, 18, 20, 22, 24, 48, and 72 h). The sorption isotherms as function of pH were obtained by adjusting the solutions to the desired pH value (1 to 7), which were left for 24 h at 45 rpm.

In both experiments, the suspensions were then centrifuged at 3500 rpm for 15 minutes and separated. A solution without europium was used as the reference. Finally, the europium uptake on solid was determined by measuring the luminescence in a Fluorolog Jobin Yvon Horiba with Xenon lamp (450 W). Quartz cells were loaded with 500 *μ*L aliquots of the supernatant from each experiment and the stock solution (as a reference). The samples were excited at 397. The europium emission spectra were recorded from 570 nm to 650 nm and the highest peaks were quantified at 590 and 610 nm [48]. An integration time 1.0 s and a wavelength increment of 0.5 nm were used. The obtained spectra were analyzed in Fluorolog software.

#### 2.4.1. Modeling Sorption Kinetics

There are different sorption models in the literature which try to explain retention of metals and radionuclides in solid substrates. These can be divided into empiric and thermodynamic models. The former is useful when it comes to establishing kinetics and sorption capacity as a first step of establishing the interaction mechanism between the metal and the solid but without taking into consideration that the surface sites strongly depend on the pH of the aqueous medium. The latter on the other hand allows a study of the dependency of the pH from the surface sites of the solid from the knowledge on the surface acidity constants and by means of the estimation of the constants of formation of surface complexes.

In the present work, four kinetic sorption models were used with the aim of explaining the obtained experimental data and determining the possible sorption mechanism of Eu^3+^ in titanium pyrophosphate. The models used were pseudo-first-order (Lagergren's equation), Elovich, pseudo-second-order, and intraparticle diffusion models.

*Pseudo-First-Order Model*. First-order kinetic model is proposed by Lagergren. This model is widely used to explain the sorption of solutes in aqueous solution [53]. The equation used is given as

(2) $$\begin{array}{c} \log\left(\;q_{e}-q_{t}\;\right)=log\left(\;q_{e}\;\right)-\frac{k^{\prime }t}{2.303}, \end{array}$$

where *q*_*e*_ is the sorption capacity at equilibrium (mg g^−1^), *q*_*t*_ is the mass adsorbed (mg g^−1^), *t* is the contact time (min), and *k*′ is the first-order sorption constant (min^−1^).

*Elovich Model*. It is a kinetic model used to describe the kinetics of heterogeneous chemical sorption of gases in solids [54]. Also, this model has been widely used to describe the chemisorption of the adsorbate by a solid in aqueous medium [55]. The model presents itself mathematically as follows:

(3) $$\begin{array}{c} \frac{\delta q_{t}}{\delta t}=\alpha \exp\left(\;-\beta {tq}_{t}\;\right), \end{array}$$

where *α* is the initial sorption velocity (mg g^−1 ^min^−1^) and *β* is the desorption constant (g mg^−1^). Other authors relate *β* to the activation energy for the chemisorption [55].

*Pseudo-Second-Order Model*. Second-order kinetic model is widely used to explain the processes of chemisorption of metals [56]. In (4), the general expression of the model is presented:

(4) $$\begin{array}{c} \frac{\delta q_{t}}{\delta t}=k_{2}{\left(\;q_{e}-q_{t}\;\right)}^{2}, \end{array}$$

where *k*_2_ is the second-order sorption velocity constant (g mg^−1^ min^−1^).

*Intraparticle Diffusion Model*. It is fractional-order kinetic model which permits establishing whether the sorption process is limited by intraparticle diffusion [57, 58]. In (5), the mathematical expression of the model is shown:

(5) $$\begin{array}{c} q_{t}=k_{i}t^{0.5}+C_{SL}, \end{array}$$

where *k*_*i*_ is the intraparticle diffusion rate (mg g^−1 ^min^−0.5^) and *C*_SL_ is a constant related to the thickness of the surface layer formed on the adsorbed. The higher the values of *C*_SL_, the greater the effect of the surface boundary layer.

#### 2.4.2. Estimation of Surface Complex

The surface complex constants for the europium species sorbed onto the titanium pyrophosphate were obtained from the sorption isotherms as function of pH using the FITEQL program and the CCM. The input data are *K*^+^, *K*^−^, and inner-layer capacitance values obtained.

In the sorption modeling, it was assumed that (a) a monodentate complex is formed by the interaction of a europium species in solution with only a single surface site and (b) a bidentate complex is formed when a europium species in solution interacts with two surface sites.

The identification of the chemical species of europium in solution, to be used as the initial input in the sorption simulation, was carried out by MEDUSA program of the Sweden Royal Institute of Technology [59].

## 3. Results and Discussions

### 3.1. Material Characterization

**Figure 1:**
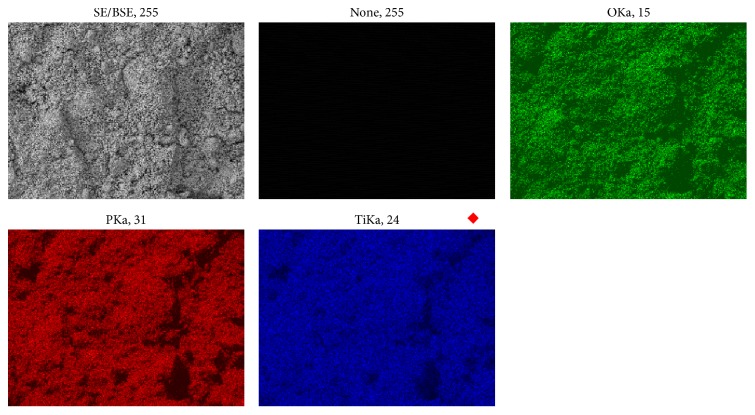
Elemental distribution of the solid obtained, where only Ti, P, and O are observed.

**Figure 2:**
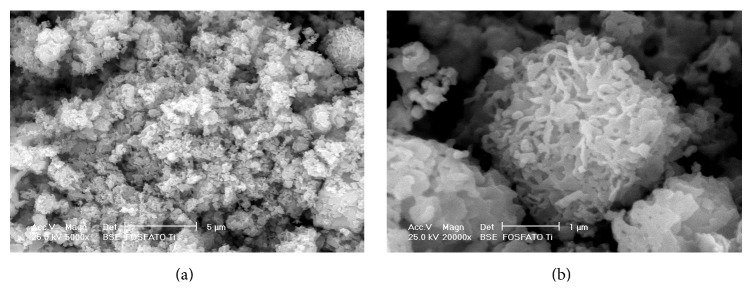
(a) Micrograph obtained at 5000x magnification, where polymorphic particles of laminar appearance could be observed (3 *μ*m). (b) Micrograph obtained at 20000x magnification, where spherical particles of irregular surface are observed (2 *μ*m).

The elementary analysis by EDS showed only oxygen, titanium, and phosphorous, and the Ti/P relationship was in agreement with the theoretical ratio in titanium diphosphate. Within the detection limits of the technique used (*Z* > 12), no contaminants were detected in the solid obtained. Additionally, Figure 1 shows a distribution map of Ti, P, and O of a sample representative of the solid. The map shows that the material has an elemental homogenous distribution.  Figure 2(a) shows the micrograph of the solid obtained at 5000x, in which polymorphic particles of laminar appearance with a size of less than 3 *μ*m can be observed.  Figure 2(b) shows the micrograph of the solid obtained at 20000x, in which particles of spherical appearance and irregular surface with an approximate size of 2 *μ*m can be observed.

**Figure 3:**
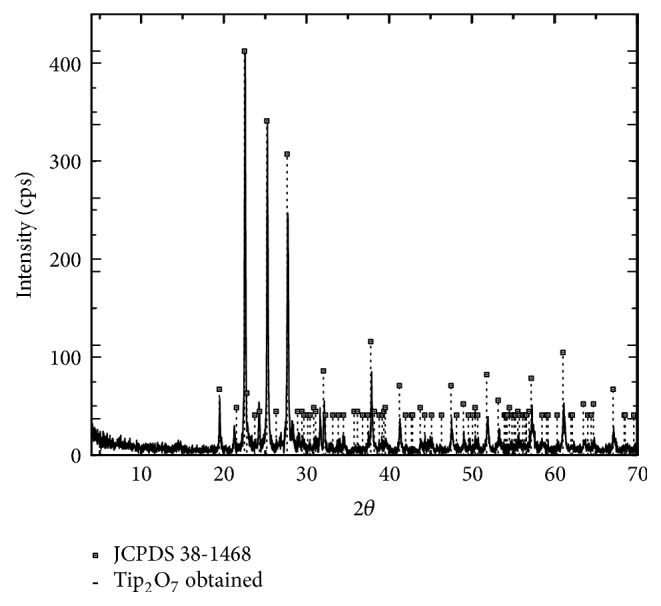
XDR spectra of the obtained material; the lines correspond to titanium pyrophosphate, pattern to JPCDS 38-1468.

Figure 3 shows XDR spectra of the sample prepared at 1073 K, in which three intense reflections located at the angle 2*θ*, 22.55, 25.25, and 27.71, identified as cubic TiP_2_O_7_ with a Pa3 (205) space group (JCPD 38-1468) can be observed. Furthermore, a slight slipping of the diffraction peaks to the right was observed, indicating loss of the superstructure. It can be explained considering that, in this work, synthesis was carried out at 1073 K during 3 h, and Norberg et al. [60] reported that at high temperatures the structure of pyrophosphate group was disordered due to having unfavorable 180 bond angles.

**Figure 4:**
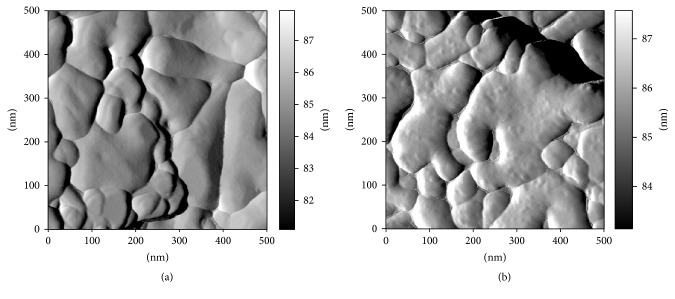
Surface imagens of titanium pyrophosphate obtained by AMF: (a) before europium sorption and (b) after europium sorption.

The results obtained from the analysis by AFM are presented in Figure 4. This shows the surface images of the titanium pyrophosphate before and after the europium sorption.  Figure 4(a) (before sorption) clearly showed various small and larger particles in the surface morphology of solid. Additionally, we observed that the surface is apparently smooth.  Figure 4(b) (after sorption) shows the influence of europium sorption on the surface morphology of titanium pyrophosphate. As can be clearly seen, the surface is fully covered with a layer of sorbed europium.

Furthermore, the results from the interpretation adsorption isotherm of N_2_ (by BET method) showed that the titanium pyrophosphate synthetized has an *A*_*s*_ of 9.0 ± 0.1 m^2 ^g^−1^.

### 3.2. Determination of Surface Properties

**Figure 5:**
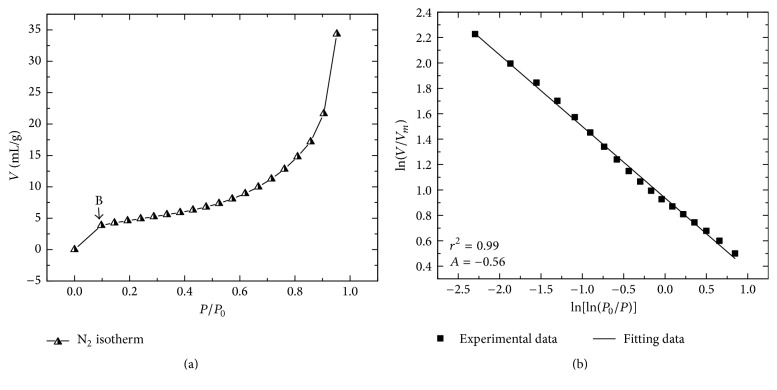
Adsorption isotherm of N_2_ of the titanium pyrophosphate; (b) adjusted results of adsorption of the isotherm N_2_.

*Surface Roughness.* Figure 5(a) shows the adsorption isotherm of N_2_. It obeys a reversible isotherm of type II that is concave at low pressure in relation to *P*/*P*_*o*_ and has originated from the physisorption of nitrogen and is typical in nonporous materials. In this figure, two perfectly defined regions can be observed. Region I (0 < *P*/*P*_*o*_ > 0.5) shows the beginning of an almost linear section (starting from point B) which indicates the ending of the monolayer. Region II (0.5 < *P*/*P*_*o*_ > 0.95) shows the beginning of the curvature of the isotherm and indicates the inflexion point of the overlap of the monolayer and the beginning of the multilayer, where *P*/*P*_*o*_ = 0.5 [61]. When *P*/*P*_*o*_ = 1, the thickness of the multilayer seems to increase without limit and the filling of the volume of the pores is caused by capillary condensation.

Figure 5(b) shows the adjusted results of the isotherm of the adsorption of N_2_ using (1). This figure was drawn up plotting ln⁡(*V*/*V*_*m*_) versus ln⁡(ln⁡*P*_*o*_/*P*). In accordance with the isotherm analysis (Figure 5(a)), it can be deduced that two fractal diameters (*D*_1_ and *D*_2_) exist, which are associated with two adsorption forces. According to Ismail and Pfeifer [50], when van der Waals attraction forces are dominant between the solid and the absorbent film, *D* = 3*A* + 3, then the surface tension of the liquid/gas is insignificant. When the capillary force is important, *D* = *A* + 3. From the equation of the adjustment of the experimental data, it has been obtained that the value of the slope *A* = −0.56 with a statistical correlation coefficient *r*^2^ = 0.99 allows establishing that *D*_1_ = 1.31 when *D* = 3*A* + 3 and *D*_2_ = 2.43 when *D* = *A* + 3. Wang et al. [44] and Cao et al. [62] suggest associating the surface of the fractal diameter as an average value of *D*_1_ and *D*_2_, the interval from 1.31 to 2.43 equaling *D*_*m*_ = 1.87. From there, it can be inferred that the surface of the synthesized solid is not rough. In the same way, Ismael and Pfeifer [50] suggest that when applying in (1) the relations *D* − 3/3 and *D* − 3, both mechanisms function simultaneously and an intermediate slope is obtained. According to Ismael and Pfeifer [50], the effect of the surface tension on the slope of the linearized equation (1) can be established from the equality *δ* = 3(1 + *A*) − 2 = −0.69, because when *D* ≥ 2 the value of *δ* should reach an absolute small value and the surface tension on the gradient would be negligible. When *δ* < 0, the surface tension is not considered insignificant. When considering the magnitude of *D*_2_, it can be established that the surface of the synthesized titanium pyrophosphate is slightly smooth when multilayers are formed at high *P*/*P*_*o*_. Likewise, the magnitude of *D* can be associated with the small number of active sites (*N*_*s*_ = 7 sites nm^−2^) [37, 38, 63] which are linked to the polar groups (OH^−^) present on the surface of the material.

*Surface Acidity Constants*. The potentiometric titration curve can be fit to determine *K*^+^, *K*^−^, and *C*. The experimental error was estimated to be ±0.2 pH units and 5% for the concentrations of H^+^ or OH^−^ added. To reduce the degrees of freedom in the determination of the surface acidity constants of titanium pyrophosphate using the FITEQL program (IV) [52], initial values were used for the parameters *N*_*s*_ and *A*_*s*_, thereby reducing the number of parameters to be solved for the equilibrium model for the system. The goodness of the fit can be determined by the ratio between the weighted sum of squares and the total degrees of freedom (WSOS/DF); for the analyzed system, this ratio was less than 20 [51, 64].

The inner-layer capacitance of the surface acidity constant must be determined. This parameter is difficult to estimate because the experimental calculation is not easy. The empirical value is often chosen as approximately 1 Fm^−2^ for mineral oxides, but in these cases, the ionic strength considered was not higher than 0.1 M [64]. Phosphate compounds are highly insulating materials and a capacitance value of 3.08 Fm^−2^ has already been used successfully for diverse phosphates with ionic strengths greater than 0.1 M [52]. For this work, we tested the previous values and found that the best fit was obtained with an inner-layer capacitance value of 3.1 Fm^−2^ for an ionic strength of 0.5 M.

**Figure 6:**
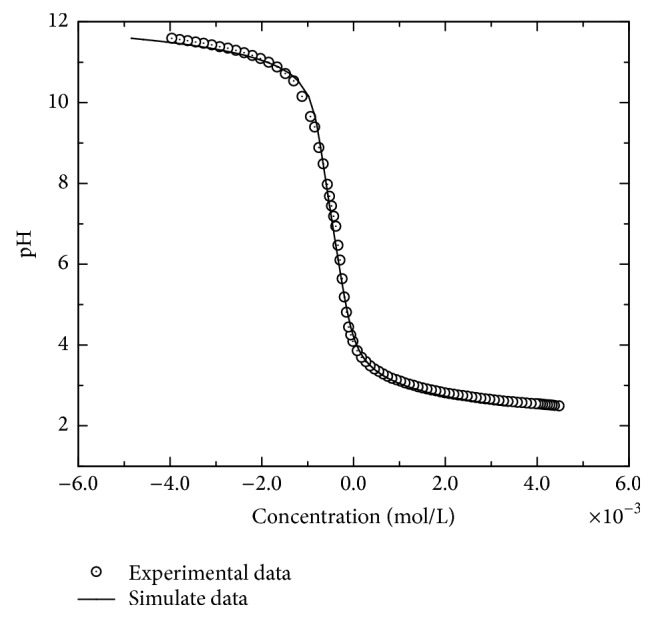
Acid/base titrations of TiP_2_O_7_ in 0.5 M potassium nitrate solution. Experimental (circle) and CCM calculated curve (line).

The experimental and simulated curves of the solid are shown in Figure 6, and Table 1 reports the obtained acidity constants. The surface acidity constants determined for titanium pyrophosphate were log *K*^+^ = 3.59 ± 0.06 and log⁡*K*^−^ = −3.90 ± 0.05; the goodness of the fit was 9. The experimental data could only be fit when considering only one surface site; these results agree with those observed by other authors in phosphates with the ≡P-OH functional group [37, 40]. As observed in Table 1, there is no significance between the values obtained for TiP_2_O_7_, ZrP_2_O_7_, and LaPO_4_. Th(PO_4_)_4_P_2_O_7_ has higher values of surface acidity constants. The differences among materials with the same functional groups are attributed to impurities, crystal structures, the synthesis method, or the background salt used to determine the ionic strength [52].

### 3.3. Sorption Experiments

Prior to the sorption experiments, the europium chemical equilibrium was determined using the MEDUSA program to determine the europium chemical species at the sorption conditions. A complimentary search was performed to find the europium species reported in the literature. The species and the formation constant obtained are reported in Table 2. Additional, the results determined using the MEDUSA program showed that, until a pH of 6, two major species are present: EuNO_3_^+^ and Eu^3+^. However, Eu(OH)_3c_ precipitated in the pH range of 6 to 11.

**Figure 7:**
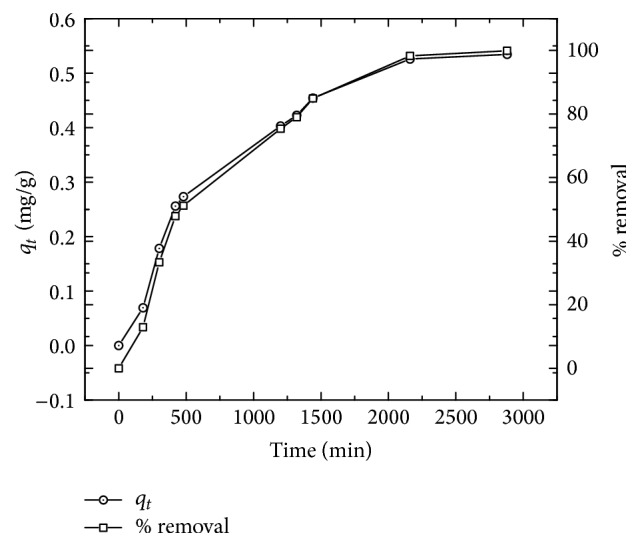
Sorption of Eu^3+^ as a function of time in the solid synthetized, using 1 × 10^−4 ^M solution of EuNO_3_, in contact with 400 mg of solid at pH 6.

Figure 7 shows the variation in quantity of europium retained in the solid over time. These results indicate that the sorption of europium occurs in two phases, during the first of which (*t* ≤ 10 h) the retention velocity is fast and grows exponentially with time, reaching its maximum velocity at times close to 10 h, during which time approximately 50% of the initial europium quantity (*q*_*t*_ = 0.28 mg g^−1^) have been retained. During the second phase the maximum sorption percentage is reached at times exceeding 35 h, where the sorption velocity diminishes significantly until reaching the thermodynamic equilibrium in which the solid is saturated and unable to retain more Eu^3+^.

#### 3.3.1. Modeling Sorption Kinetics

The analysis of the experimental data of the sorption of europium according to time was carried out using the empiric models previously described.

**Figure 8:**
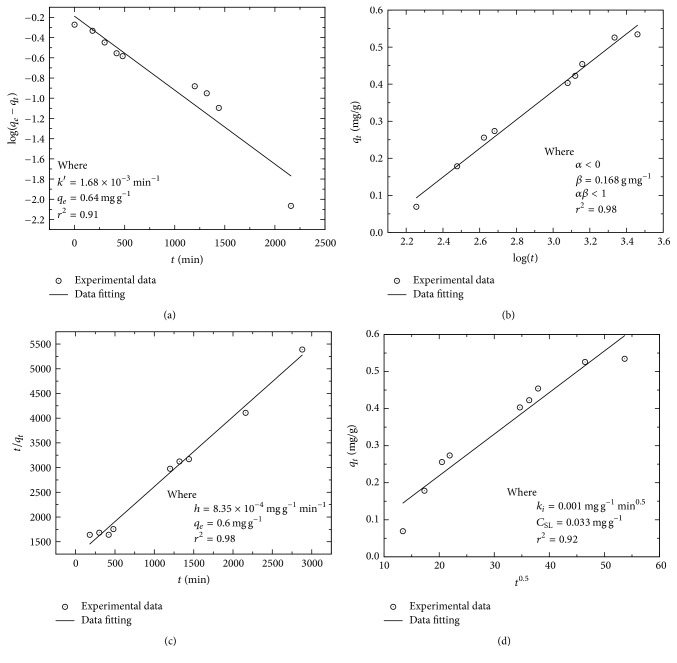
(a) Experimental data fit of the pseudo-first-order model (Lagergren's equation); (b) experimental data fit using the linearized equation of the Elovich model, *q*_*t*_ = *α* + 2.303*β*log⁡(*t*); (c) experimental data fit using the linearized equation of pseudo-second-order model proposed by Ho et al. (2005), *t*/*q*_*t*_ = *q*/*h* + (1/*q*_*e*_)*t*; (d) experimental data fit using the linearized equation of the intraparticle diffusion model.

Figure 8(a) shows the results of the adjustment of the experimental data using the linearized equation of the Elovich model [48, 49]. The model parameters and the statistical coefficient were obtained plotting *q*_*t*_ versus log⁡*t*. As can be seen in the figure, the adjustment of the experimental data has a statistical coefficient *r*^2^ of 0.98. The slope and ordinate of the adjustment equation allowed an estimation of the model parameters, during which it was determined that *α* < 0 and *β* = 0.168 mg g^−1^. These results show that the Elovich model explains the experimental data mathematically as it counts on a good correlation. Nevertheless, the model assumes that the product of *αβ* ≫ 1, since in our case *αβ* < 0, it can be established that the sorption process of Eu^3+^ on solid cannot be explained phenomenologically with the Elovich model.

Figure 8(b) shows the results of the adjustment of the experimental data using the pseudo-first-order model. This analysis was carried out plotting log⁡(*q*_*e*_ − *q*_*t*_) versus *t*. As can be seen in the graphic, the statistical correlation obtained (*r*^2^) was 0.91. The kinetic parameters *k*′ and *q*_*e*_, whose values are estimated as 1.68 × 10^−3 ^min^−1^ and 0.64 mg g^−1^, respectively, were calculated using the adjustment equation.

Figure 8(c) shows the results of the adjustment of the experimental data using the pseudo-second-order model and using the linear adjustment equation proposed by Ho et al. [56], which was obtained by plotting *t*/*q*_*t*_ versus *t*. With the adjustment equation, it was estimated that *h* = 8.35 × 10^−4^ mg g^−1^ min^−1^, *q*_*e*_ = 0.6 mg g^−1^, and *k*_2_ = 1.8 × 10^−3^ g mg min^−1^. The obtained correlation coefficient was 0.98. This correlation showed that the model explains the experimental data adequately; likewise the magnitudes of the kinetic parameters are congruent from a physical point of view, which indicates that the retention process of europium on titanium pyrophosphate is possibly achieved by chemisorption.

Finally, Figure 8(d) shows the adjustment of the experimental data using a model of fractional order (intraparticle diffusion model). In this case, the adjustment analysis was carried out by plotting *q*_*t*_ versus *t*^0.5^. The results showed that *k*_*i*_ = 0.001 mg g^−1 ^min^−0.5^ and *C*_SL_ = 0.033 mg g^−1^, with a *r*^2^ = 0.92. This correlation shows that the sorption process of Eu is not limited by intraparticle diffusion albeit it suggests that it can be influenced by diffusive phenomena: diffusion in the liquid/solid interphase or diffusion on the surface. Different authors have pointed out that when the adjustment is high and gives a straight line as a result this indicates that the process is exclusively controlled by intraparticle diffusion; otherwise, when the adjustment data respond to more than one adjustment line, this indicates that the sorption process is influenced by more than one diffusive phenomenon [53, 65].

From the analysis of the adjustment results of the different models, it is possible to determine that the pseudo-second-order model can be used to explain the sorption kinetics for europium in the titanium pyrophosphate. It should be noted that the sorption capacity of the solid studied is like that observed in other phosphates with P_2_O_7_ groups. Studies such as those carried out by Wang et al. [34] show that the nanostructured titanium pyrophosphates and the isomorphous substitution of Zr^4+^ by Ti^4+^ result in an enhancement of sorption capacity of actinides. Other materials, such as the novel SiO_2_-ZrO_2_-calcium alginate aerogels [66] or graphene [67], have a higher actinide retention capacity than the solid studied, but their structural stability has not been demonstrated when subjected to intense fields of thermal and ionizing radiation.

Furthermore, the tested models indicate that the sorption of europium on the solid occurs through the diffusion of the metallic ion in the solid/liquid interphase and on the surface of the solid, subsequently through the interaction of the ion with the surface sites or functional groups of the titanium pyrophosphate, which favors the formation of strong bonds between the europium and the material. This retention mechanism has been observed in different heavy metals such as Cr, Cd, and Pb [68].

#### 3.3.2. Estimation of Surface Complex Surface

**Figure 9:**
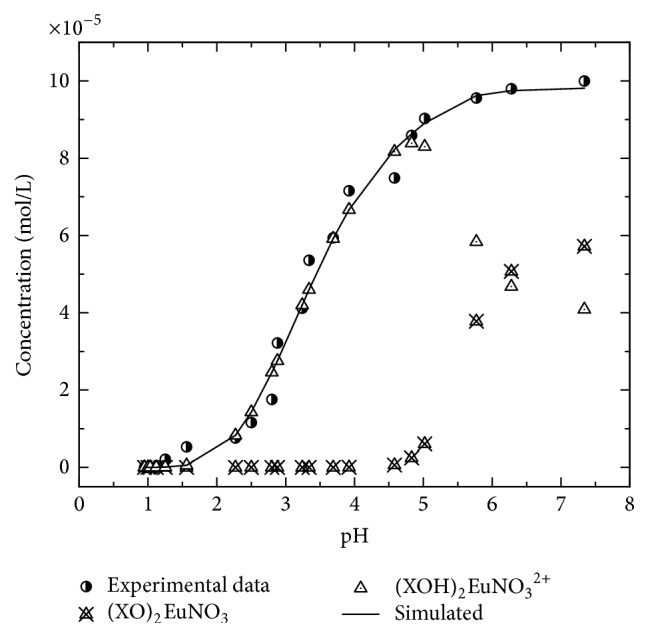
Eu^3+^ sorption modeling on titanium pyrophosphate and calculated curves.

Figure 9 shows the europium sorption isotherm of Eu^3+^ onto titanium pyrophosphate, and the sorption edge spreads between pH = 2 and pH = 5.5, which indicated that the sorption process involves more than one surface complex. Under the experimental conditions, 50% of the europium was sorbed approximately at pH values between 2 and 3.5 and the rest of the Eu^3+^ was sorbed in the interval of 3.5–6.

To fit the sorption curve, *K*^+^, *K*^−^, and *C* values were fixed at the values determined above. During the experimental data analysis, the error was estimated to be ±0.2 pH units and 5% for the total europium concentration. We used this information to identify the surface complexes that are involved in the retention of europium by titanium diphosphate. Figure 9 presents the sorption modeling. The results obtained from the experimental data show the probable existence of two bidentate complexes on the surface. In the pH range of 2 to 4.5, two surface sites are required to form the bidentate complex (≡XOH)_2_EuNO_3_^2+^ with the elimination of one hydrogen; in a similar way, in the pH range of 4.5 to 7, two surface sites are required (≡XO^−^) to form a bidentate complex with EuNO_3_^2+^ ((≡XO)_2_EuNO_3_).

Therefore, the europium sorption mechanisms in titanium pyrophosphate result from the interaction of a ≡XOH_2_^+^ surface site (pH < 3.3) and a ≡XO^−^ surface site (pH > 3.3) with EuNO_3_^2+^ and the formation of the inner-sphere bidentate surface complexes. For pH values, greater than 7, the mechanism that reduces the concentration of the europium in the solution is the chemical precipitation of the condensate species onto the surface.

Table 3 shows the values for the sorption constants obtained from this research of Eu^3+^ on titanium pyrophosphate and also those for the Eu^3+^ complex formed in similar phosphates. The result obtained in this work for the first surface europium complex is similar to that reported by Finck [38] for the ZrO complexed with europium at 308 K, which could be attributed to (≡TiOH)EuNO_3_^2^; however, the value for the P_2_O_7_ complex is different. Finck [38] reports a value of −3.2 ± 0.3, but Drot et al. [40] report a value of 0.94 ± 0.14 for the P_2_O_7_ complex in the Th(PO_4_)_4_P_2_O_7_. Drot's value is in good agreement with the value obtained for the second Eu (III) complexation constant in this work: 1.2 ± 0.5. This research determined that the value for the Eu-PO_4_ complex in the same compound was −2.23 ± 0.13; similarly, Drot [37] found a value of −3.0 ± 0.3 for the complex of Eu with (PO_4_).

## 4. Conclusions

The proposed technique allows for the synthesis of a pure titanium pyrophosphate as confirmed by the analytical techniques. The surface behavior as function of pH shows the formation of ≡XOH_2_^+^ species at pH values lower than 3.3 and ≡XO^−^ at higher pH values. Only two species, Eu^3+^ and EuNO_3_^2+^, were present in the solution in the sorption pH range of 2 to 5.5.

It has been established that the surface characteristics of TiP_2_O_7_ are similar to ZrP_2_O_7_ and other phosphates with the ≡P_2_O_7_ and ≡P-OH functional groups. However, the modeling of the sorption curves determined that the mechanism of europium sorption on the solid is first due to external and surface diffusion and second due to chemisorption related to the possible formation of two types of inner-sphere bidentate surface complexes. The stability constants of the formed complexes show the feasibility of using titanium pyrophosphate as an engineering barrier in the containment of radioactive waste.

## Figures and Tables

**Table 1:** Surface acidity constants of titanium pyrophosphate.

Solid	Capacitance (Fm^−2^)	log⁡*K*^+^	log⁡*K*^−^	Functional group	Ref.
TiP_2_O_7_	3.08	3.59 ± 0.06	−3.90 ± 0.05	P_2_O_7_	This work
ZrP_2_O_7_	3.08	3.2	−4.2	P_2_O_7_	[37, 38]
LaPO_4_	3.08	3.6	−5.4	PO_4_	[39]
Th_4_(PO_4_)_4_P_2_O_7_	3.08	6.5	−7.8	P_2_O_7_	[40, 41]

**Table 2:** Equilibrium constants of the chemical species of europium in 0.5 M KNO_3_ solution.

Chemical equilibrium equation	log *β*, FI = 0.5 M, *T* = 298 K	Reference
Eu^3+^ + H_2_O *↔* Eu(OH)^2+^	−8.53	[42]
Eu^3+^ + 2H_2_O *↔* Eu(OH)_2_^+^	−17.31	[42]
Eu^3+^ + 3H_2_O *↔* Eu(OH)_3_	−26.35	[42]
Eu^3+^ + NO_3_^−^*↔* Eu(NO_3_)^2+^	0.29	[43]
Eu^3+^+ CO_3_^2−^*↔* Eu(CO_3_)^+^	6.11	[42]
Eu^3+^ + 2CO_3_^2−^*↔* Eu(CO_3_)_2_^−^	10.45	[44]

**Table 3:** Eu (III) sorption equilibria onto titanium pyrophosphate and other compounds and associated constants.

Surface complex	log *K*	Compound	Reference
≡XOH + Eu^3+^ + NO_3_^−^*↔* (≡XOH)EuNO_3_^2+^	5.8 ± 0.1	TiP_2_O_7_	This work
≡XOH + Eu^3+^ + NO_3_^−^*↔* (≡XO)_2_EuNO_3_	1.2 ± 0.5	TiP_2_O_7_	This work
2(≡ZrOH) + Eu^3+^ *↔* (≡ZrOH)_2_Eu^3+^	6.25 ± 0.3	ZrP_2_O_7_	[38]
2(≡POH) + Eu^3+^ + NO_3_^−^*↔* (≡PO)_2_Eu + 2H^+^	−3.2 ± 0.3	ZrP_2_O_7_	[38]
2≡XOH + Eu^3+^ + NO_3_^−^*↔* ≡(XOH)_2_EuNO_3_^2+^	7.49 ± 0.05	ZrP_2_O_7_	[40, 41]
2≡XOH + Eu^3+^ + NO_3_^−^*↔* ≡(XO)_2_EuNO_3_ + 2H^+^	0.94 ± 0.14 (P_2_O_7_)	Th(PO_4_)_4_P_2_O_7_	[37, 40, 41]
2≡XOH + Eu^3+^ + NO_3_^−^*↔* ≡(XO)_2_EuNO_3_ + 2H^+^	−2.23 ± 0.13 (PO_4_)	Th(PO_4_)_4_P_2_O_7_	[37, 40, 41]
2≡XOH + Eu^3+^ + NO_3_^−^*↔* ≡(XO)_2_EuNO_3_ + 2H^+^	−3.0 ± 0.3 (PO_4_)	Zr_2_O(PO_4_)_2_	[37, 40]
2≡XOH + Eu^3+^ + NO_3_^−^*↔* ≡(XO)_2_EuNO_3_ + 2H^+^	0.31 ± 0.5 (oxo)	Zr_2_O(PO_4_)_2_	[37, 40]
